# *Ascophyllum nodosum* Extract Biostimulant Processing and Its Impact on Enhancing Heat Stress Tolerance During Tomato Fruit Set

**DOI:** 10.3389/fpls.2020.00807

**Published:** 2020-06-25

**Authors:** Nicholas Carmody, Oscar Goñi, Łukasz Łangowski, Shane O’Connell

**Affiliations:** ^1^Plant Biostimulant Group, Shannon Applied Biotechnology Centre, Institute of Technology Tralee, Tralee, Ireland; ^2^Research and Development Department, Brandon Bioscience, Tralee, Ireland

**Keywords:** tomato (*Lycopersicon esculentum*), plant biostimulants, *Ascophyllum nodosum* extracts, abiotic stress tolerance, heat stress, flowering, carbohydrates, heat shock protein

## Abstract

The application of biostimulants derived from extracts of the brown seaweed *Ascophyllum nodosum* has long been accepted by growers to have productivity benefits in stressed crops. The impact of the processing method of the *A*. *nodosum* biomass is also known to affect compositional and physicochemical properties. However, the identification of the mechanisms by which processing parameters of *Ascophyllum nodosum* extracts (ANEs) affect biostimulant performance in abiotically stressed crops is still poorly understood. In this study, we performed a comparative analysis of two carbohydrate-rich formulations derived from *A*. *nodosum*: C129, an ANE obtained at low temperatures through a gentle extraction and the novel proprietary PSI-494 extracted under high temperatures and alkaline conditions. We tested the efficiency of both ANEs in unstressed conditions as well as in mitigating long-term moderate heat stress in tomato (*Lycopersicon esculentum*, cv. Micro Tom) during the reproductive stage. Both ANEs showed significant effects on flower development, pollen viability, and fruit production in both conditions. However, PSI-494 significantly surpassed the heat stress tolerance effect of C129, increasing fruit number by 86% compared to untreated plants growing under heat stress conditions. The variation in efficacy was associated with different molecular mass distribution profiles of the ANEs. Specific biochemical and transcriptional changes were observed with enhanced thermotolerance. PSI-494 was characterized as an ANE formulation with lower molecular weight constituents, which was associated with an accumulation of soluble sugars, and gene transcription of protective heat shock proteins (HSPs) in heat stressed tomato flowers before fertilization. These findings suggest that specialized ANE biostimulants targeting the negative effects of periods of heat stress during the important reproductive stage can lead to significant productivity gains.

## Introduction

In the last decade, reports show that climate change has significantly impacted on overall food security due to a reduction in viable land areas, global yields of many staple crops, and an increase in both biotic and abiotic stresses (Ainsworth and Ort, 2010; FAO, 2016). In the field, abiotic stresses can occur in isolation or together to impact phenotypical, physiological, biochemical, and/or molecular aspects of crop development. Heat stress, can have significant implications on important plant activities, such as seed germination, plant development, photosynthesis, and reproduction, which leads to reductions in plant growth and crop yield (Rieu et al., 2017; Nadeem et al., 2018). The most recent IPCC report predicts a global increase of more than 4°C until the end of the century and a more frequent occurrence of severe heat waves (Change, 2014). Under these conditions, it has been predicted that a 1°C increase in global temperature could decrease the production of important commodity crops between 4.1 and 6.4% (Zhao et al., 2017). An assessment of heat stress risk at a global level for four key crops (wheat, maize, rice, and soybean) suggests that the global warming impact on agriculture production would not only occur in sub-tropical and tropical regions, but also in important agricultural regions such as Eastern China, the Northern United States, South-Western Russian Federation, and Southern Canada (Teixeira et al., 2013).

A key factor in the successful implementation of agronomic strategies to enhance crop thermotolerance is a better understanding of the mechanisms by which heat stress alters plant metabolism and leads to crop yield losses. While heat stress typically occurs when temperatures rise 5–15°C above the optimum for plant growth, the impact of high temperatures on crop yield is defined by the intensity, duration, and rate of the temperature change. Generally, two types of high temperature stresses can be distinguished. A short period of very high temperatures (e.g., >15°C above optimum temperature) is generally referred as heat shock and can cause extensive damage on crop plants by affecting vital physiological and metabolic functions such as enhanced respiration, photoinhibition of photosystem II (PSII), increase in membrane fluidity, accumulation of reactive oxygen species (ROS), changes in carbohydrate partitioning, or protein denaturation (Fragkostefanakis et al., 2015; Fahad et al., 2017; Nadeem et al., 2018). However, exposure to moderately elevated temperatures (e.g., 5–10°C above optimum growth temperature) would require a longer exposure (i.e., multiple days) to obtain similar effects (Mesihovic et al., 2016). It is important to mention that the susceptibility of individual crops to a specific heat stress regime would also vary with the developmental stage of the plant. While plants at vegetative stage are able to maintain basic activities and to minimize the injuries derived from long-term moderate heat stress, reproductive development tends to be more affected under these conditions. Moreover, it has been observed in both monocot and dicot species that male gametophytes (pollen grains) are even more susceptible to damage from heat stress than their female counterparts in both long-term moderate and extreme heat stress. Therefore, the number and health of the reproductive organs will influence fruit set in heat stressed conditions, a critical phase for realizing yield potential (Mesihovic et al., 2016; Rieu et al., 2017).

Several solutions for providing crop thermotolerance include specialty crop inputs, selective plant breeding, or genetic modification approaches. The exogenous application of proline in heat stressed chickpea seedlings coupled an improved content of chlorophyll and antioxidant compounds with a significant improvement in the activities of enzymes of carbon fixation and sucrose metabolism (Kaushal et al., 2011). Other studies have shown how the foliar application of salicylic acid (SA) or phosphite in young plants reduced the adverse effects of extreme heat stress regimes through enhanced photosynthesis or accumulation of osmoprotectants (Khan et al., 2013; Xi et al., 2020). Breeding programs to obtain thermotolerant cultivars have focused on traits such as better photosynthetic rate, pollen viability, or fruit set under high temperatures. However, the development of new thermotolerant varieties through plant breeding is expensive and time-consuming (Chapman et al., 2012; Fahad et al., 2017). Another way to increasing yield under heat stress is based on the generation of genetically modified (GM) thermotolerant crops. Numerous plant species have shown increased thermotolerance through the enhancement of synthesis of heat shock proteins (HSPs), using various transgenic approaches (Grover et al., 2013; Gerszberg and Hnatuszko-Konka, 2017). HSPs are the first line of defense against heat stress damage acting as molecular chaperones in order to reduce or even prevent denaturation or aggregation of proteins and increasing the refolding of protein structure (Jacob et al., 2017). These evolutionarily conserved proteins affect a broad array of cellular processes and are grouped into five classes in plants, according to their molecular weight: HSP100, HSP90, HSP70, HSP60, and small heat-shock proteins (sHSPs; Reddy et al., 2016).

Plant biostimulants have been gaining increased attention during the last number of years, due to the growing interest of scientists, private industry, and growers in integrating these products into their armory of environmentally friendly tools that can assist in securing improved crop performance (Du Jardin, 2015; Yakhin et al., 2017; Rouphael and Colla, 2020). Globally, the biostimulant market is forecast to expand at a growth rate of 12.3% CAGR (Compound Annual Growth Rate) from 2019 to 2027 and it is expected to reach US$ 5.5 billion by 2027 (Transparency Market Research, 2019). After several years of negotiations among the European institutions, the new Fertilising Products Regulation (FPR) (EU) 2019/1009 was published in the Official Journal of the EU on 25th June 2019, recognizing plant biostimulants as a distinct category of agricultural inputs. Under the new regulation: “A plant biostimulant shall be a EU fertilizing product, the function of which is to stimulate plant nutrition processes independently of the product’s nutrient content with the sole aim of improving one or more of the following characteristics of the plant and the plant rhizosphere: (a) nutrient use efficiency, (b) tolerance to abiotic stress, or (c) quality traits” (Regulation (EU) 2019/1009, 2019).

In the plant biostimulants field, the positive effects of seaweed extracts have been extensively demonstrated (Sangha et al., 2014), turning them into the fastest growing product category in the global plant biostimulant market (Markets and Markets, 2019). However, it is important to note that seaweed extract biostimulants are not a homogenous category of products. Seaweed extracts vary depending on the family and species of seaweed used for manufacture (e.g., brown, green, or red), the source of the seaweed raw material and the process used for extraction (Craigie, 2011; Fletcher et al., 2017). The brown seaweed *Ascophyllum nodosum* has long been accepted by growers in the international market to have superior performance as compared to biostimulants made from other seaweeds. *Ascophyllum nodosum* extract (ANE) biostimulants have been shown to improve plant vigor, increase root development, enhance chlorophyll synthesis, promote earlier flowering, enhance fruit set and uniformity of fruit, reduce pod shatter, delay senescence, and enhance tolerance to abiotic stress (Sangha et al., 2014; Łangowski et al., 2019; Shukla et al., 2019). The impact of processing of the *A. nodosum* raw material on an ANE biostimulant product is also known to affect compositional and bioactivity-related parameters (Goñi et al., 2016, 2018). However, little attention has been paid to the identification of the mechanisms by which processing parameters of ANEs can affect their biostimulant performance in heat stressed crops.

After potato, tomato (*Lycopersicon esculentum*) is considered the most valuable vegetable crop grown globally. Although tomatoes normally grow in tropical, subtropical, and warm temperate climates, which facilitate longer growing seasons, losses of up to 70% can be seen in areas affected by summers with unusually high temperatures (Sato et al., 2002). Both extreme temperatures and prolonged periods of moderately elevated temperatures can impact different plant activities leading to reductions in fruit set or fruit yield (Mesihovic et al., 2016). Different tomato plant cultivars growing under chronic mild heat stress showed that pollen release, pollen viability, and anther morphology were major limiting factors for optimum fruit set (Sato et al., 2000; Müller et al., 2016; Xu et al., 2017). One of the main biochemical parameters that influenced pollen viability and development of young tomato fruits during heat stress periods was an optimal carbohydrate metabolism (Pressman et al., 2002; Firon et al., 2006; Li et al., 2012; Zhou et al., 2017). Furthermore, Fragkostefanakis et al. (2016) showed that heat stress response and thermotolerance in tomato developing pollen was linked to the accumulation of heat stress-induced chaperones, such as *HSP101.1*, *HSP70.9*, *HSP17.7C-Cl*, and other protective metabolites.

In this study, we performed a comparative analysis of two carbohydrate-rich biostimulant formulations derived from *A*. *nodosum*: C129, an ANE obtained at low temperatures, and the novel proprietary PSI-494 extracted under high temperatures and alkaline conditions through a targeted plant signal induction (PSI) approach to formulation development. We tested the efficacy of both ANEs in unstressed conditions as well as in mitigating long-term moderate heat stress in tomato during the reproductive stage. Evaluating specific phenotypical, physiological, biochemical, and molecular markers associated with enhanced thermotolerance, we revealed the distinct effect of ANEs obtained through different extraction methods and how it can be linked to their different molecular weight distribution profiles. Here, we show for the first time that the judicious application of specialized ANE biostimulants can target the negative effects of periods of high temperatures during the important reproductive stage and solve specific plant productivity challenges.

## Materials and Methods

### Chemicals

The carbohydrate rich fraction of *A. nodosum*, crude enzymatic mixture, and the two ANEs; C129 and PSI-494 complex were provided by Brandon Bioscience (Tralee, Ireland). All chemical reagents and dextran standards were purchased from Sigma-Aldrich (Arklow, Ireland). The primers were purchased from Eurofins Genomics (Ebersberg, Germany).

### Plant Material and Growth Conditions

Tomato seeds (*L*. *esculentum*, cv. Micro Tom) were purchased from Liscahane Nurseries (Tralee, Ireland). Seeds were surface sterilized with sodium hypochlorite for 1 min before being thoroughly rinsed with distilled water. Seed were set in plug trays using a growth medium composed of compost:vermiculite:perlite (5:1:1). On day 22 seedlings were transferred to 1 L pots [same growth medium as previous with the addition of 2 g calcium carbonate lime and 1 g of slow releaser fertilizer containing N/P_2_O_5_/K_2_O (7/7/7, w/w/w)]. Plants were raised in a growth room at a temperature of 27/22 ± 2°C with 16 h of daylight and 8 h of night and 80 ± 5% relative humidity (RH) under a light intensity of 120 μmol m^−2^ s^−1^ in a complete randomized block design. Plants were irrigated with 1.5 L of water per tray twice a week in order to create equal soil moisture conditions in all pots. Temperature and relative moisture content were recorded regularly with a portable USB data logger (Log32TH, Dostmann electronic GmbH).

### ANE Biostimulant Treatment Application and Heat Stress Conditions

Two formulations (C129 and PSI-494) obtained from a carbohydrate rich fraction of *A. nodosum* using two different extraction methods were applied to plants as ANE biostimulant treatments. The initial carbohydrate rich fraction was isolated using selective solvents according to Rioux et al. (2007). The C129 extract was obtained after treating the carbohydrate rich extract with a crude enzymatic mixture with carbohydrate depolymerizing activity at low temperature (<30°C). PSI-494 was produced using a proprietary extraction at high temperatures and alkaline conditions from the same carbohydrate rich fraction. Both ANE biostimulants possessed a very low macronutrient content with N:P:K values of 0.3–0.4:0.1–0.2:2–3% w/w. Prior to the application of heat stress, the ANE biostimulants were applied by foliar spray at a dilution of 0.106% (w/v) on 105-day-old plants with significant presence of flowers at early stages of pollen development (4–8 mm young buds). Water was applied as a control. After 3 days, plants of all three groups (control, C129 and PSI-494) were exposed to moderate heat stress for 14 days in a growth chamber (31/24°C with 16 h of light and 8 h of darkness and 80 ± 5% RH under a light intensity of 120 μmol m^−2^ s^−1^). To minimize the influence of any positional effect, the relative position of the pots was changed every other day. Tomato pollination was aided using an electric toothbrush twice a week when plants began to flower. After the heat stress period, the plants were placed back in the growth room and ANE treatments were applied again as foliar spray at 0.106% (w/v). Control plants were sprayed with equal volume of distilled water. Recovery stage after heat stress was maintained for 1 week under unstressed conditions to obtain 129-day-old plants at fruit set stage. A two-spray application program before and after the stress period was based on current farmer practice for the use of ANEs and previously published by Goñi et al. (2018). Leaf and flower tissue were sampled in 122-day-old plants after being subjected to moderate heat stress for 14 days. Similar tomato plants were selected and grown under unstressed conditions for 122 days. ANE biostimulants and control treatments were applied by foliar spray as described above to evaluate growth promoting effects on non-heat stressed tomato plants. Leaf and flower sampling points for unstressed plants also corresponded to 122-day-old plants. All leaf and flower samples were collected 2 h after the end of the light period to avoid any influence of plant day-night cycle on soluble sugar profiles, snap-frozen in liquid nitrogen, ground and kept in −80°C until further analysis.

### Chemical Composition and Structural Analysis of ANE Biostimulant Treatments

Dried ANE samples were placed inside a furnace at 600°C for 6 h in order to obtain and quantify the ash content. Total sugars were quantified according to Rioux et al. (2007). Total polyphenol content was determined spectrophotometrically following the method of Goñi et al. (2018). The content of unidentified organic components was calculated by difference to the total organic amount. The molecular weight (M_w_) distribution of carbohydrates from different samples was analyzed using high performance size exclusion chromatography-refraction index detector (HPSEC-RID). The HPSEC Shimadzu system consisted of a system controller CBM-20A, a solvent delivery module LC-20 AD, an online degasser DGU-20A5, an autosampler SIL-20ACHT, a refraction index detector (Varian Prostar 350 RID), and an LC workstation. HPSEC analysis was performed using 4 PL aquagel-OH MIXED-H columns in tandem (8 μm, 300 × 7.5 mm; Agilent). The mobile phase (0.1 M NaAc/0.1 M Na_2_SO_4_ buffer pH 7.8) was used as isocratic elution at room temperature. The flow rate and injection volume were set to 1 ml min^−1^ and 40 μl, respectively. M_w_ values were calculated from the measured retention times through a calibration curve made with dextran standards.

### Evaluation of Plant Height, Photosynthetic Performance, Total Flower and Fruit Number

Plant height, total number of flowers and photosynthetic performance were evaluated at the end of the heat stress period. The sampling point for unstressed plants corresponded to 122-day-old plants. Regarding the photosynthesis parameters, PQ-SPAD (relative chlorophyll content), Φ_II_ (quantum yield of PSII) and Φ_NPQ_ (quantum yield of non-photochemical exciton quenching) were evaluated using a MultispeQ device (Kuhlgert et al., 2016). Fruit set was evaluated at the end of the recovery stage in heat-stressed plants. The sampling point for unstressed plants corresponded to 129-day-old plants. A developing fruit was considered as a small, ripening fruiting body that had displaced the tomato flower which was containing it.

### Evaluation of Pollen Viability

To conduct pollen viability analysis, one flower per plant at anthesis stage was collected by removal of the flower and bud using sterile forceps at the end of the heat stress period. The sampling point for unstressed plants corresponded to 122-day-old plants. Pollen viability was determined according to Paupière et al. (2017). Briefly, flowers were placed in a tube with 500 μl of germination solution [1 mM KNO_3_, 3 mM Ca(NO_3_)_2_, 0.8 Mm MgSO_4_, and 1.6 Mm H_3_BO_3_] and 20 μl of Alexander dye. Samples were left overnight at room temperature to allow for consistent staining of the pollen grains. Viable pollen was stained purple by the Alexander dye, while non-viable pollen was stained green. Pollen number was quantified using a Neubauer chamber hemocytometer. The recorded results were then transformed into the number of each type of pollen per flower and the results were expressed as percentage of viable pollen.

### Sucrose, Glucose and Fructose Content in Plant Tissues

The levels of sucrose, glucose, and fructose were determined by HPAEC-PAD using a Carbopac PA-1 column and expressed as mg g^−1^ FW in leaf and flower tissue following the method of Goñi et al. (2018). These soluble sugars were measured in samples collected either at the end of the heat stress period or for 122-day-old unstressed plants. The measured results were expressed as difference of heat stressed samples (Untreated, C129, and PSI-494) with respect to the unstressed control.

### RNA Extraction and RT-qPCR

Total RNA was isolated from about 50 mg of frozen ground flower material by Plant/Fungi Total RNA Purification Kit (Norgen Biotek, Canada) following the manufacturer’s instructions. RNA was treated with RNase-Free DNase I Kit (Norgen Biotek, Canada) in order to remove efficiently genomic DNA contamination. RNA concentration and purity was measured in a μDrop™ Plate RNA using a Varioskan Flash instrument (Fisher Scientific). Expression analysis of *HSP101.1* (*Solyc03g115230*), *HSP70.9* (*Solyc11g020040*), and *HSP17.7C-Cl* (*Solyc06g076520*) genes was performed by RT-qPCR using a Roche LightCycler® 96 System (Roche, UK) and a LightCycler® RNA Master SYBR Green I one-step kit (Roche, UK) according to the manufacturer’s instructions. The expression level of the tomato *ACTIN2* (*Solyc01g104770.2*) gene was used as the reference gene. 2^−ΔΔCT^ was used to quantify normalized gene expression. The primers sequences used were as follows:

***HSP101.1***: forward 5'-ACCCGATCAGATTGCGGAAG-3' and reverse 5'-GAACCAGTTGGTTGCTGTGG-3'.
***HSP70.9***: forward 5'-GAGCTCAAGGATGCCATTTC-3' and reverse 5'-CAGATGATCCAGTTGTACCAG-3'.
***HSP17.7C-Cl***: forward 5'-ATGGAGAGAAGCAGCGGTAA-3' and reverse 5'-ATGTCAATGGCCTTCACCTC-3'.
***ACTIN2***: forward 5'-TCTTGAAGCGTTTTAAAAGATGGC-3' and reverse 5'-TCACCAGCAAATCCAGCCTT-3'.

### Statistical Analysis

Phenotypic assessment of plants was done in three independent plant trials, with six plants per treatment group and condition (18 independent biological replicates). The flower and leaf samples collected per independent plant trial were pooled for further analysis (three independent pooled biological samples for every plant tissue sample). Chemical and structural analysis of ANEs was performed on a minimum number of three biological replicates. Photosynthetic parameters were measured in one leaf at a central position for every plant (18 independent biological replicates) using three technical replicates per biological replicate. For biochemical and molecular analysis, at least three biological replicates of each treatment and condition were performed using the plant samples described above and three technical replicates per biological replicate were used. Statistics were evaluated with Sigma Plot 12 and Statgraphics Centurion XVI software. The differences in the chemical or structural analysis of ANEs were analyzed using *t*-test at *p* ≤ 0.05. The effect of ANE treatments on plant soluble sugar content and *HSPs* gene expression were analyzed with the one-way analysis of variance (ANOVA) by Tukey’s HSD test at *p* ≤ 0.05. The rest of the plant data was compared by using two-way ANOVA, with Tukey’s HSD test at *p* ≤ 0.05. Where the interaction between the two factors condition (unstressed and heat stressed) and ANE treatment (AxB) was significant, data were subjected to one-way ANOVA, comparing all ANE treatments with each other within the same growth condition. Where AxB interaction was not significant, the effect of condition and ANE treatment was evaluated separately, comparing the respective means through *t*-test (condition) or one-way ANOVA Tukey’s HSD test (ANE treatment) at *p* ≤ 0.05. The application of all parametric tests was performed after checking the data normality (Shapiro-Wilk test) and equal variance assumptions. Unless stated otherwise, all data are expressed as average ± standard error (SE). Details of the individual sample size for each analysis are mentioned in the table and figure legends.

## Results

### Compositional and Structural Analysis of ANE Biostimulants

**Figure 1:**
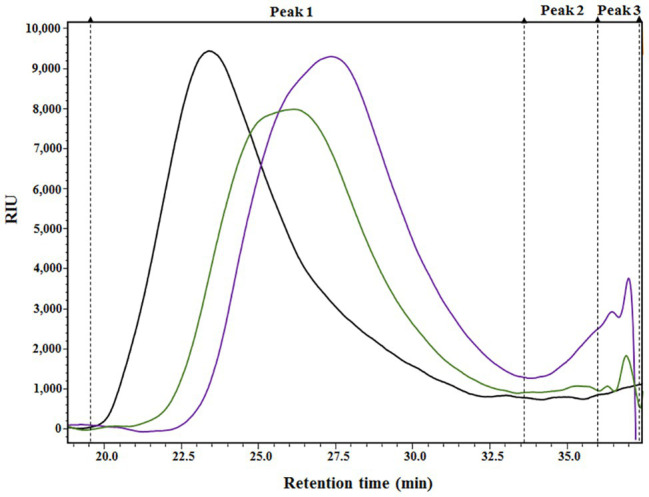
HPSEC-RID analysis of the initial carbohydrate rich substrate and the generated *Ascophyllum nodosum* extract (ANE) biostimulants. Black chromatogram (AN substrate); green chromatogram (C129); purple chromatogram (PSI-494). The three main peaks were integrated and are shown in the chromatograms with dashed lines.

The results presented in Table 1 provide a compositional evaluation of the two ANE biostimulants by determining the levels of some key components, such as ash, total carbohydrates, and polyphenols. C129 and PSI-494 were primarily composed of carbohydrates and ash. Both ANE biostimulants differed by <2% in the amount of total carbohydrates (*p* = 0.278). The analysis of polyphenols, determined as phloroglucinol equivalents, indicated that both ANEs contained very low amounts of this component on a dry weight basis and there were not statistically significant differences between them (*p* = 0.374). As it can be observed in the HPSEC-RID chromatograms (Figure 1), the initial carbohydrate rich fraction used as substrate was composed of a homogenous 555.31 kDa peak which generated three carbohydrate peaks for both ANE biostimulants. These peaks were characterized as three groups of different molecular weights (Table 2). The C129 formulation, which was extracted gently at low temperatures was composed of a mix of molecules ranging between 2,881.47 and 1.28 kDa with a very high representation (97%) of molecules with an average M_w_ of 212.12 kDa. However, PSI-494 extracted at high temperatures using a proprietary formulation process was characterized as a product with lower M_w_ carbohydrates. Molecules ranging between 1.29 and 3.24 kDa were significantly more abundant than those observed in C129 (8.82 vs. 1.76%) and the average M_w_ of the PSI-494’s main peak was 1.8-fold smaller than that characterized in the ANE biostimulant obtained at low temperatures.

**Table 1:** Compositional analysis of two ANE biostimulant treatments.

Component % (w/w)^1^	ANE biostimulant treatment	
	C129	PSI-494
Ash^2^	32.10 ± 0.67	35.81 ± 0.87^*^
Total carbohydrates	64.81 ± 0.60	63.52 ± 0.55
Polyphenols	0.65 ± 0.08	0.55 ± 0.06
Other organics	2.44 ± 0.21	0.12 ± 0.03^**^

1 Data are the means ± SE (number biological replicates; *n* = 3).

2 ANEs chemical compositional analysis is expressed with respect to their dry content.

* Difference was significant at *p* ≤ 0.05 (*t*-test).

** Difference was significant at *p* ≤ 0.001 (*t*-test).

**Table 2:** Molecular weight distribution of two ANE biostimulant treatments expressed as the average M_w_ of the main peaks, the M_w_ corresponding to the interval of the whole peak or the relative peak area.

Treatment	# Peak	Peak properties^1^			
		Average M_w_ (kDa)	Start M_w_ (kDa)	End M_w_ (kDa)	Area (%)^2^
C129	1	212.12 ± 0.25	2,881.47 ± 2.34	3.11 ± 0.07	97.04 ± 0.56
	2	2.32 ± 0.07	3.11 ± 0.07	1.67 ± 0.03	1.29 ± 0.04
	3	1.45 ± 0.04	1.67 ± 0.03	1.28 ± 0.03	0.47 ± 0.02
PSI-494	1	117.67 ± 0.18	2,676.67 ± 2.15	3.24 ± 0.11	91.19 ± 0.45^*^
	2	1.87 ± 0.05	3.24 ± 0.11	1.68 ± 0.03	6.65 ± 0.07^*^
	3	1.44 ± 0.03	1.68 ± 0.03	1.29 ± 0.04	2.17 ± 0.03^*^

1 Data are the means ± SE (number biological replicates; *n* = 4).

2 The peak area was calculated in *percentage* over the total *area* of all *peaks*.

* Differences between the respective peak area values of both ANEs were significant at *p* ≤ 0.001 (*t*-test).

### Effect of Heat Stress and ANEs on Height of Tomato Plants

**Figure 2:**
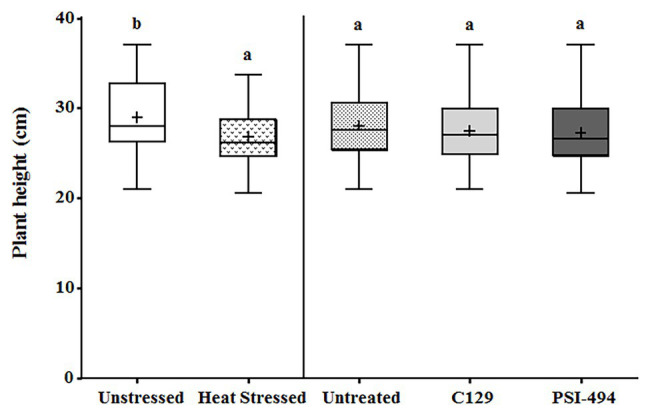
Effect of heat stress and ANEs on height of tomato plants. Data were measured in 122-day-old plants and subjected to two-way ANOVA and Tukey’s HSD test for evaluating the differences among means at *p* ≤ 0.05. Since there was no significant AxB interaction, the effect of condition and ANE treatment was evaluated separately, comparing the respective means. Different letters indicate statistical differences for *p* ≤ 0.05 based on *t*-test (condition) or one-way ANOVA Tukey’s HSD test (ANE treatment). The vertical line is used to visually separate the evaluation of the effect of condition and ANE treatment. The horizontal line through the box and the cross represent the median and mean value, respectively. Number of biological replicates = 18.

The plant height was recorded from the start of the stem (at soil level) to the dorsal flowering body (highest point of the plant) in 122-day-old tomato plants. The two-way ANOVA test revealed that in conjunction both parameters (condition × ANE treatment) had no significant effect (*p* = 0.698; Table 3). The heat stressed plants showed an overall statistically significant decrease of plant height compared to the unstressed group (unstressed: 29.42 cm vs. heat stressed: 26.46 cm; *p* = 0.014; Figure 2). However, the effect of the different ANEs on this parameter was not statistically significant (*p* = 0.650) with respect to the control.

**Table 3:** Source of variance for height, reproductive, and photosynthetic parameters of tomato plants grown at two temperature conditions and treated with two ANE biostimulants.

Source of variance	Plant height	Flower number	Pollen viability	Fruit number	PQ-SPAD	Φ_II_	Φ_NPQ_
Condition (A)	*	***	***	*	ns	***	***
ANE treatment (B)	ns	*	***	***	ns	ns	ns
AxB	ns	ns	***	*	ns	*	*

ns, non-significant; *significant at *p* ≤ 0.05, **significant at *p* ≤ 0.01, and ***significant at *p* ≤ 0.001, respectively.

### Effect of Heat Stress and ANEs on Reproductive Development Parameters of Tomato Plants

**Figure 3:**
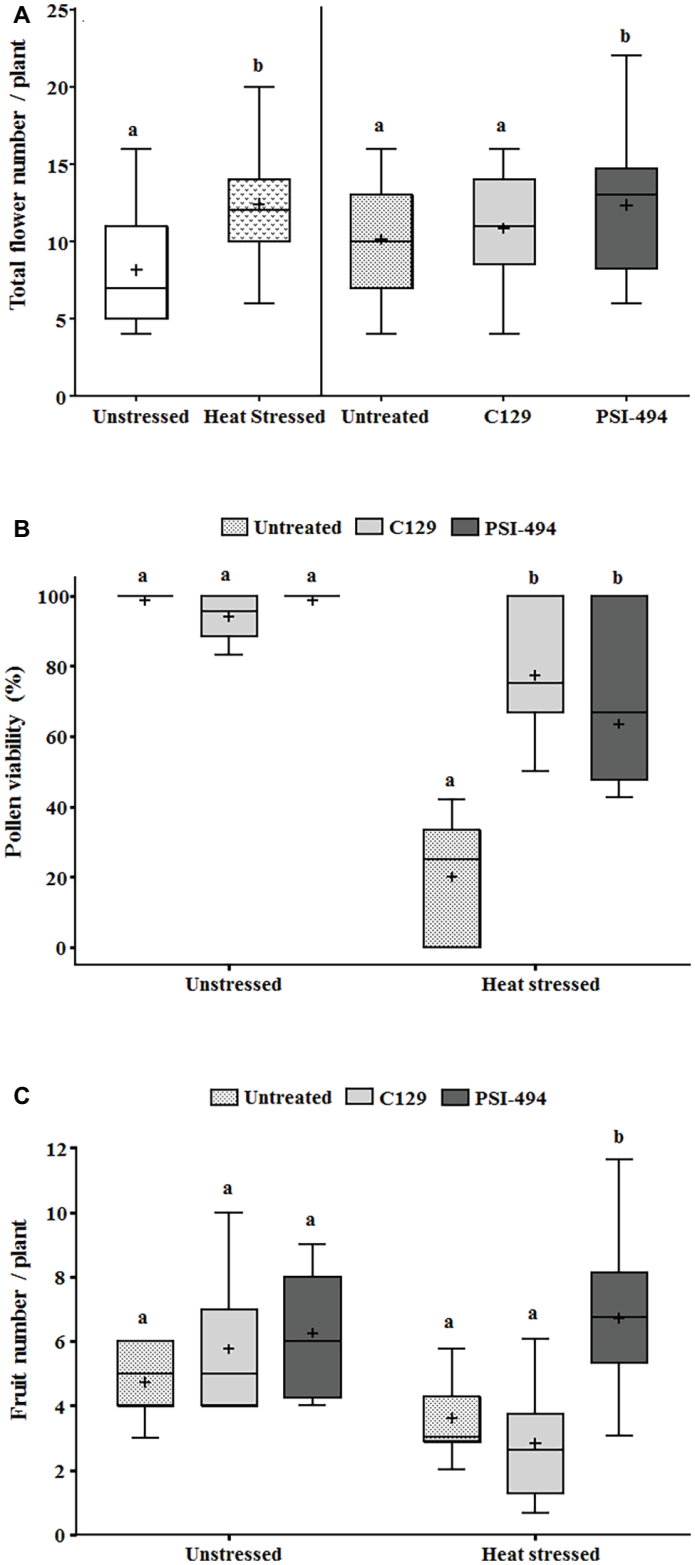
Effect of heat stress and ANEs on reproductive parameters of tomato plants. **(A)** Total flower number; **(B)** pollen viability; and **(C)** fruit number. Total flower number and pollen viability were measured in 122-day-old plants and fruit number in 129-day-old plants. Data were subjected to two-way ANOVA and Tukey’s HSD test for evaluating the differences among means at *p* ≤ 0.05. Since there was no significant AxB interaction for total flower number, the effect of condition and ANE treatment was evaluated separately, comparing the respective means. Different letters indicate statistical differences for *p* ≤ 0.05 based on *t*-test (condition) or one-way ANOVA Tukey’s HSD test (ANE treatment). The vertical line is used to visually FIGURE 3separate the evaluation of the effect of condition and ANE treatment. However, since interaction AxB was significant for pollen viability and fruit number, data were subjected to one-way ANOVA, comparing all ANE treatments with each other within the same growth condition (unstressed or heat stressed). In this case, different letters indicate statistical differences for *p* ≤ 0.05 based on Tukey’s HSD test. The horizontal line through the box and the cross represent the median and mean value, respectively. Number of biological replicates = 18.

In order to evaluate how long-term moderate heat stress and ANE biostimulant treatments affected the reproductive stage of tomato plants, three different developmental parameters were evaluated (total flower number, pollen viability, and fruit number). As can be observed in Table 3, the two-way ANOVA test showed that the interaction between factors was not significant for the total flower number (*p* = 0.754). On the contrary, there was a significant increase in the number of flowers per plant in both treatments between unstressed (7.89) and heat stressed (12.55) plants (*p* ≤ 0.001). When these differences were examined in detail in terms of the ANE treatment group, there were significant differences between the treated and untreated plants (*p* = 0.024). Interestingly, those plants treated with PSI-494 showed the highest absolute values of total flower number (11.98) compared to the control (8.95; Figure 3A).

Tomato pollen viability was compromised when plants were exposed to moderate heat stress for 14 days (31/24°C; Figure 3B). When a two-way ANOVA test was run, it was found that all three parameters (condition, ANE treatment, and condition × ANE treatment) were highly significant (*p* ≤ 0.001; Table 3). Therefore, all data were subjected to one-way ANOVA, comparing all ANE treatments to each other under both growing conditions. One hundred twenty-two-day-old plants growing under unstressed conditions (27/22°C day/night) had pollen viability over 94%. Only the application of C129 led to a small but not statistically significant decrease with respect to control (−4.56%; *p* = 0.196). Although viable pollen was reduced by 80% in untreated plants growing under heat stress, our results also showed that C129 and PSI-494 significantly increased this parameter between 3.2 and 4.4 times compared to the control. However, no statistically significant differences were observed between both ANE treatments under heat stress conditions (Figure 3B).

Fruit number was quantified in 129-day-old tomato plants to determine how the effect of both heat stress damage during the recovery period and the second ANE application may impact on this yield related parameter (Figure 3C). When a two-way ANOVA test was applied, it was found that there was a statistically significant interaction between condition and ANE treatment (*p* = 0.011; Table 3). Therefore, data of ANE treatments were examined using one-way ANOVA and results indicated that tomato plants grown under unstressed conditions and sprayed twice with C129 and PSI-494 increased their fruit number by 22 and 33%, respectively. However, this improvement in fruit set was not statistically significant (*p* = 0.289). PSI-494 did significantly increase fruit number by 86% compared to the untreated group in heat stressed plants (*p* ≤ 0.001). This parameter did not show significant differences in stressed tomato plants treated with C129 (*p* = 0.455; Figure 3C).

### Effect of Heat Stress and ANEs on Photosynthetic Parameters of Tomato Plants

**Figure 4:**
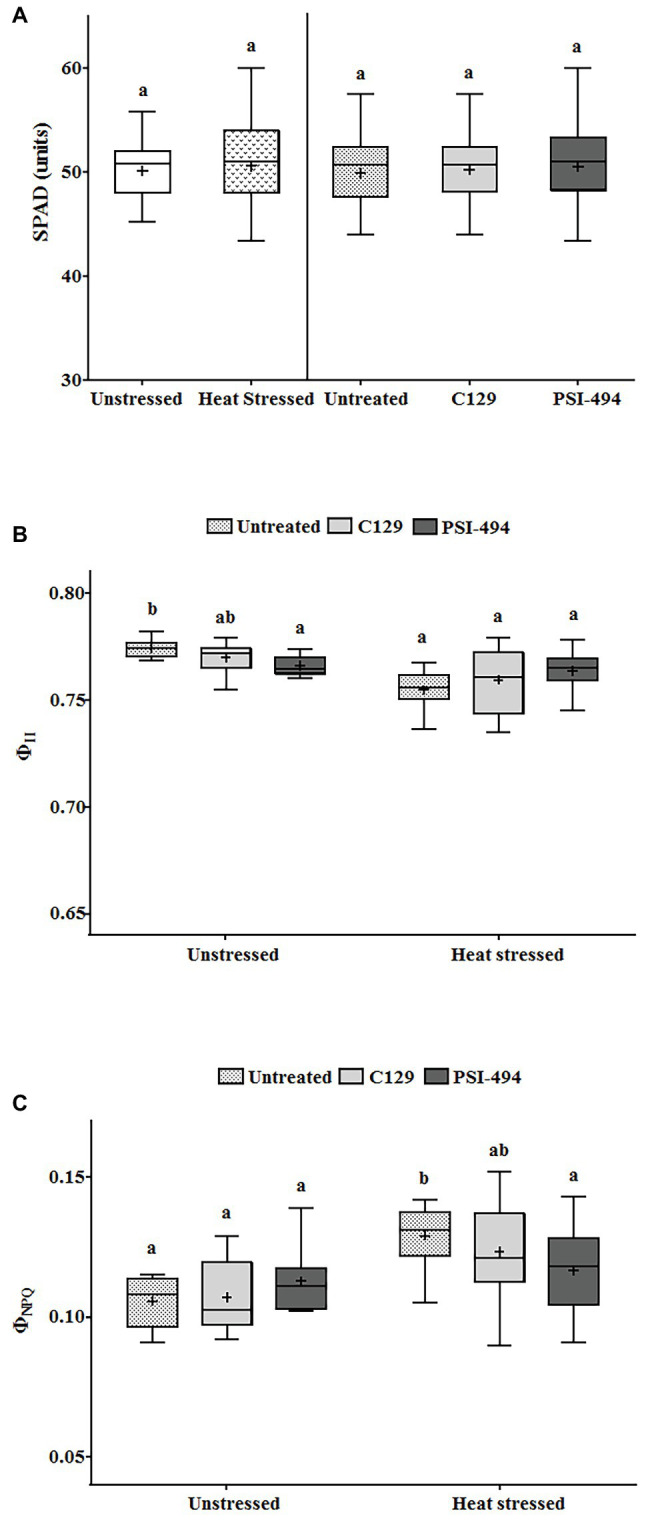
Effect of heat stress and ANEs on photosynthetic parameters of tomato plants. **(A)** PQ-SPAD; **(B)** Φ_II_; and **(C)** Φ_NPQ_. Data were measured in 122-day-old plants and subjected to two-way ANOVA and Tukey’s HSD test for evaluating the differences among means at *p* ≤ 0.05. Since there was no significant AxB interaction for PQ-SPAD, the effect of condition and ANE treatment was evaluated separately, statistical differences for *p* ≤ 0.05 based on *t*-test (condition) or one-way ANOVA FIGURE 4comparing the respective means. Different letters indicate Tukey’s HSD test (ANE treatment). The vertical line was used to separate visually the evaluation of the effect of condition and ANE treatment. However, since interaction AxB was significant for Φ_II_ and Φ_NPQ_, data were subjected to one-way ANOVA, comparing all ANE treatments with each other within the same growth condition (unstressed or heat stressed). In this case, different letters indicate statistical differences for *p* ≤ 0.05 based on Tukey’s HSD test. The horizontal line through the box and the cross represent the median and mean value, respectively. Number of biological replicates = 18.

Statistical analysis showed that the interaction between condition and treatment was not significant for the PQ-SPAD parameter (*p* = 0.579; Table 3). This parameter, which measures leaf chlorophyll content, was not significantly affected either by moderate heat stress (*p* = 0.893) or the application of both ANE treatments (*p* = 0.681; Figure 4A).

When Φ_II_ was analyzed using a two-way ANOVA test, it was found that two parameters (condition and condition × ANE treatment) showed statistically significant differences (*p* ≤ 0.001 and *p* = 0.012, respectively; Table 3). While this photosynthetic parameter was more affected by long moderate heat stress than PQ-SPAD, it was only reduced by 2.25% compared to untreated unstressed plants. Although all treatments in both the unstressed and heat stressed groups had quantum yield values of PSII of over 0.750 (Figure 4B).

The parameter Φ_NPQ_ was used to evaluate the effects of heat stress or ANE application on the efficiency of PSII in the energy dissipation in tomato chloroplast. A two-way ANOVA analysis found that both condition (heat stress) and condition × ANE treatment had a significant effect on Φ_NPQ_ (*p* ≤ 0.001 and 0.039, respectively; Table 3). Under heat stress conditions, the small reduction of Φ_II_ was associated with an increase of Φ_NPQ_ by 21.69% compared to untreated unstressed conditions. However, when data of all treatments were analyzed using one-way ANOVA, similar values were observed in treated and untreated unstressed plants. Interestingly, this parameter was reduced by 9% in heat stressed plants sprayed with PSI-494 compared to control stressed conditions (*p* = 0.034; Figure 4C).

### Effect of Heat Stress and ANEs on Soluble Sugar Content in Tomato Leaves and Flowers

**Figure 5:**
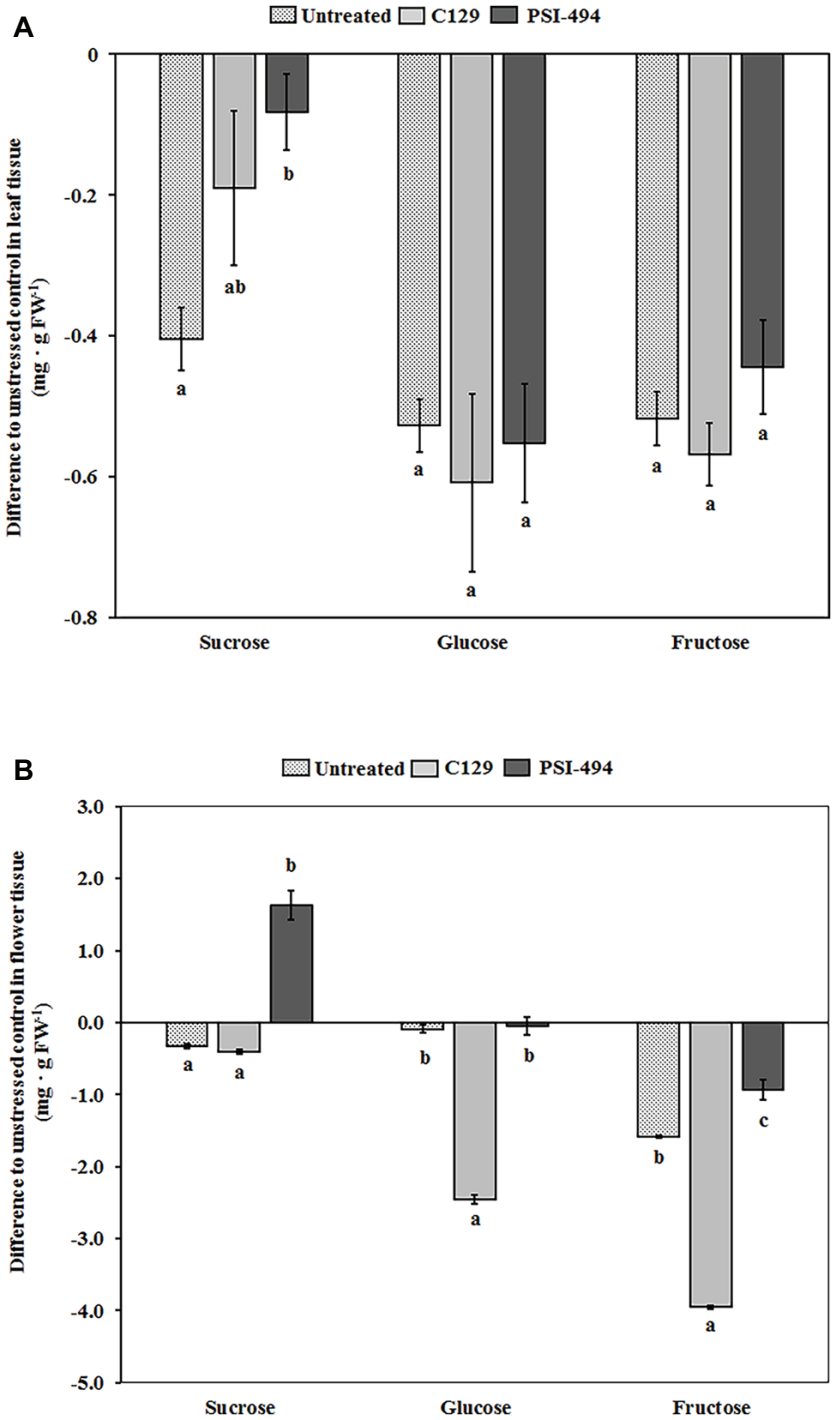
Effect of heat stress and ANEs on endogenous soluble sugars of tomato plants. The levels of glucose, fructose, and sucrose were determined by HPAEC-PAD in **(A)** leaf tissue and **(B)** flower tissue of 122-day-old plants. Measured results were expressed as difference of the three heat stressed group samples (Untreated, C129, PSI-494) with respect to the unstressed control. The straight line at the “0” level represents the unstressed control and histograms represent the absolute variations of heat stressed plants. Different letters within the same soluble sugar indicate statistically significant differences between the treatments based on one-way ANOVA Tukey’s HSD test at *p* ≤ 0.05. Number of biological replicates = 4.

The soluble sugar content in both leaf and floral tissues of 122-day-old plants was quantified by HPAEC-PAD. Our analysis revealed that the content of sucrose, glucose, and fructose in the leaf tissue of unstressed untreated plants was 1.06, 0.98, and 1.05 mg g^−1^ FW, respectively. Figure 5A shows that the content of these soluble sugars in leaf tissue decreases in untreated heat stressed plants compared to unstressed control plants. Interestingly, plants treated with PSI-494 showed a significantly lower decrease of sucrose content in foliar tissue relative to the unstressed healthy controls (−8%; *p* = 0.047) compared to that quantified in untreated stressed plants or plants treated with C129 (−38 and −18%, respectively). The foliar content of glucose and fructose was similar in untreated and ANE-treated heat stressed plants but was between 42 and 62% lower than that observed in leaf tissue of untreated unstressed plants (Figure 5A).

In the flower tissue of unstressed untreated tomato plants, fructose and glucose became the major soluble sugars (5.59 and 2.98 mg g^−1^ FW) and sucrose the minor sugar (1.47 mg g^−1^ FW). As it can be observed in Figure 5B, the content of sucrose, glucose, and fructose in untreated heat stressed flowers decreased by 22, 3, and 28%, respectively, with respect to that observed in the same tissue of unstressed control plants. However, heat stressed tomato plants treated with PSI-494 accumulated the highest content of soluble sugars in the flowers compared to the stressed control. The ANE biostimulant extracted at high temperatures was able to ameliorate the decrease of glucose and fructose (−1 and −17%, respectively). In addition, it induced a statistically significant accumulation of sucrose (11%; *p* ≤ 0.001) with respect to those values measured in untreated unstressed plants. However, flowers of heat stressed plants treated with C129 showed the lowest measured values, decreasing their content of endogenous sucrose, glucose, and fructose by 27, 82, and 71%, respectively, with respect to the unstressed control (Figure 5B).

### Effect of Heat Stress and ANEs on Expression of *HSP* Genes in Tomato Flowers

**Figure 6:**
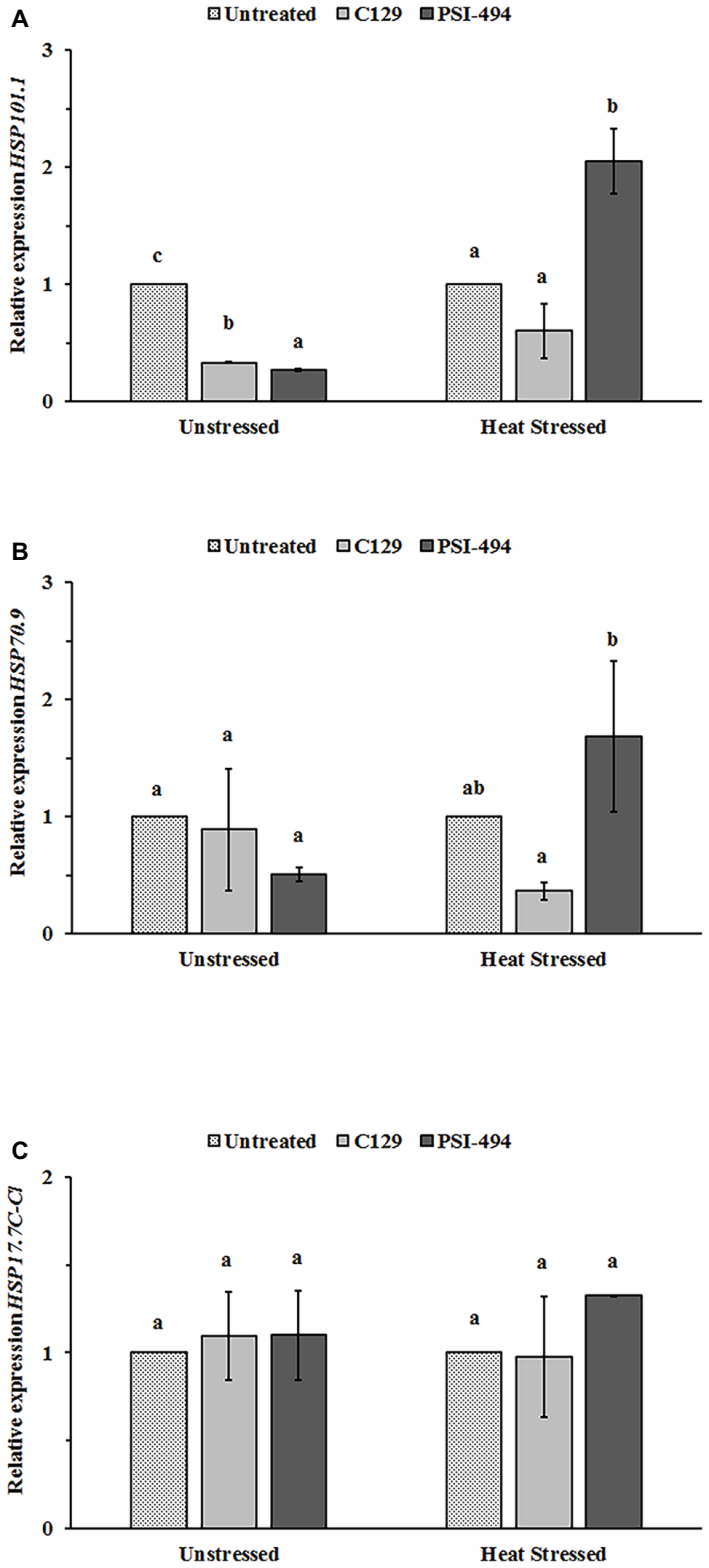
Effect of heat stress and ANEs on *HSPs* gene expression in tomato flowers. **(A)** *HSP101.1*; **(B)** *HSP70.9*; and **(C)** *HSP17.7C-Cl*. Data were measured in 122-day-old plants and expressed as the relative fold-change with respect to the *ACTIN2* (*ACT2*) gene expression levels. Different letters within the same growth condition indicate statistically significant differences between the treatments based on one-way ANOVA Tukey’s HSD test at *p* ≤ 0.05. Number of biological replicates = 3.

In order to examine whether C129 and PSI-494 biostimulants affected the regulation of three stress-protective HSPs (*HSP101.1*, *HSP70.9*, and *HSP17.7C-Cl*) at transcriptional level in tomato flowers, relative changes in gene expression were analyzed by RT-qPCR in unstressed and heat stressed 122-day-old plants (Figure 6). When the unstressed group was examined, it was found that both ANE biostimulants decreased *HSP101.1* expression level between three and four times with respect to the control (*p* ≤ 0.001; Figure 6A). However, no statistically significant differences were observed in the expression levels of *HSP70.9* and *HSP17.7C-Cl* between ANE-treated and -untreated unstressed plants (Figures 6B,C). Different effects were found when the expression of these three *HSPs* genes was examined in flower tissue grown under moderate heat stress. The application of the PSI-494 caused a significant upregulation within *HSP101* and *HSP70.9* expression levels by 2.05‐ and 1.68-fold with respect to the stressed control. *HSP17.7C-Cl* transcript level was 1.23 times higher in flowers of stressed plants treated with PSI-494 although this was not significant (*p* = 0.160). Conversely, the relative gene expression of the tested *HSPs* was similar or slightly downregulated in flowers of heat stressed plants treated with C129 vs. the control (Figure 6).

## Discussion

As part of the current global climate change, ambient temperatures are rising at a considerable rate and heat waves are becoming more frequent and severe. In many crop plants, including both monocots and dicots, elevated temperatures lead to reduced yield, which is alarming considering global food security (Change, 2014; Nadeem et al., 2018). Therefore, ensuring high yield under more unfavorable conditions is one of the greatest challenges of this century. Current knowledge shows that the plant heat stress response is highly complex, and heat tolerance should not be regarded as a single trait. Likewise, it has become clear that the focus on heat stress tolerance now has to be redirected from the vegetative to reproductive tissues due to their higher sensitivity to environmental fluctuations and their direct relationship with fruit production (Mesihovic et al., 2016; Rieu et al., 2017). Interestingly, although the utilization of plant biostimulants has proved popular for their ability to enhance abiotic stress tolerance (Van Oosten et al., 2017; Shukla et al., 2019), research literature describing the utilization of these crop inputs to provide heat stress tolerance is scarce. The available literature is mostly focused on plant species at vegetative stage (Kauffman et al., 2007; Zhang and Ervin, 2008; Botta, 2012). Therefore, it is important to expand the current knowledge to other relevant crops during the reproductive phase to build credibility and acceptance in agricultural practice.

### Processing Parameters of ANE Biostimulants Influence Thermotolerance in Tomato Plants

In this study, we found significant differences in the ability of two ANE biostimulants derived from the same carbohydrate rich fraction to induce tomato plant tolerance to moderate heat stress during the reproductive stage of the growth cycle. Two foliar applications of PSI-494, extracted under high temperatures and alkaline conditions, significantly enhanced the number of flowers, pollen viability, and fruit set compared to untreated control. However, the observed improvement in pollen viability in heat stressed plants treated with an ANE extracted at low temperatures (C129) did not translate to subsequent higher fruit set. Although the positive effects of seaweed biostimulants were initially correlated with phytohormone-like activity or the presence of compounds such as betaines (Craigie, 2011), growing evidence highlights seaweed carbohydrates as essential components in eliciting plant biostimulant activity (Goñi et al., 2020). According to the obtained chemical compositional data, more than 97% of C129 and PSI-494 corresponded to similar amounts of mineral content and carbohydrates. Therefore, as observed in a previous study on drought stress tolerance (Goñi et al., 2018), the results of the ANE biostimulant compositional analysis and heat stressed plant phenotype data were not correlated. However, processing conditions did have a significant role on one key structural parameter of carbohydrates related to M_w_ distribution. It was evident from the HPSEC-RID analysis that the proprietary extraction method used to generate PSI-494 was more successful in reducing the average M_w_ of carbohydrates extracted from the *A*. *nodosum* biomass. The most significant differences between PSI-494 and C129 were a smaller main carbohydrate peak and the higher relative abundance of secondary peaks. Previous studies have confirmed that low M_w_ polysaccharides and oligosaccharides from seaweeds were able to stimulate efficiently abiotic stress tolerance in several crop species (Liu et al., 2013; Wu et al., 2016; Singh et al., 2017; Li et al., 2018; Salachna et al., 2018). Our results would also suggest that there is a link between the lower molecular size of carbohydrates inside ANEs and enhanced heat stress tolerance in tomato plants at reproductive stage.

### Impact of ANEs on Phenotypic and Physiological Markers of Heat Stress Tolerance

This research was focused on challenging flowering tomato plants with temperatures several degrees above their optimal conditions for anthesis and fruit development for multiple days (22–26°C; Luo, 2011). As opposed to heat shocks applied for short time periods (e.g., a few hours), this experimental design was considered to be more representative of naturally occurring stress conditions in the field. As moderate heat stress regimes might significantly affect the function of vegetative tissues and impair further reproductive cell functions, we also evaluated plant height and photosynthetic activity. However, the data presented demonstrate that moderate heat stress had little effect on overall tomato stem growth. In line with this, Zhou et al. (2017) found that mild heat stress conditions (36/28°C day/night) applied in two tomato cultivars at anthesis stage for 7 days did not have significant effects on plant growth compared to unstressed conditions. Likewise, neither C129 nor PSI-494 had any statistically significant effect on plant height, suggesting that this vegetative trait may not be sensitive enough to describe the effects of ANE biostimulants.

Reduced fertility is a common problem associated with heat and has been found to be caused by high temperatures around meiosis (8–9 days before anthesis) and fertilization (2–3 days after anthesis) in various species (Mesihovic et al., 2016; Rieu et al., 2017). Therefore, the heat stress regime for 14 days was designed to study its effect not only on pollen development but also on the progamic phase and implications in respect to some heat sensitive reproductive traits in tomato. The results confirmed the harmful effects of moderate heat stress on pollen viability in tomato plants, which is in agreement with other studies (Sato et al., 2000; Pressman et al., 2002; Paupière et al., 2017; Xu et al., 2017). The application of ANE treatments did not have a substantial effect on plants grown under unstressed conditions (27/22°C day/night temperatures); however, there was a significant difference in the pollen viability between treated and untreated plants when heat stress was applied (31/24°C day/night temperatures). Plants treated with C129 and PSI-494 had higher pollen viability percentages than the untreated group after heat stress exposure, without significant differences in the efficacy of both ANE biostimulants. Although previous studies have highlighted the positive impact of seaweed extracts on different pollination parameters of high value crops such as grape or eggplant (Sabir, 2015; Pohl et al., 2019), no research to date has demonstrated the protective effect of ANEs on pollen under heat stress conditions. Furthermore, it has been previously described in different plant species that different stress types can stimulate precocious flowering and further seed production as an emergency response to highly unfavorable environmental conditions (Takeno, 2016). This stimulatory response would explain the increase in flowers in the heat stressed plants. Although promoted flowering has previously attributed to other commercial ANE biostimulants (Abubakar et al., 2012; Pohl et al., 2019), there was only a significant increase in flower number in tomato plants treated with PSI-494. These results highlight that despite the ANE biostimulants being manufactured from the same raw material their processing conditions can affect their ability to provide phenotypic and physiological benefits.

Fruit set interacts with other well-known heat sensitive traits determined before fertilization happened. For example, no positive correlations between either pollen viability or fruit set was found in ANE-treated plants under unstressed conditions, suggesting that male fertility was not a limiting factor for reproduction under optimal temperature growth conditions. Similar to the results of Xu et al. (2017) with several tomato cultivars, we also observed a clear positive correlation between fruit set and pollen viability in untreated plants under moderate heat stress. However, pollen viability values were not able to explain the measured differences in fruit set of ANE-treated plants subjected to long-term mild heat stress. Tomato plants treated with C129 decreased fruit number 1 week after heat stress with respect to control, PSI-494 stimulated a significant increase of this yield-related parameter. The fruit set value could be the result of the synergistic interaction between enhanced pollen viability and higher flower number observed after spraying PSI-494. The large differences observed in tomato plants treated with this ANE biostimulant suggest that other specific biochemical and molecular markers associated with enhanced thermotolerance could be also involved.

While heat stress can induce significant changes in photosynthetic apparatus of the plant (Mathur et al., 2014), chlorophyll spectrophotometric and fluorometric measurements only showed small changes after 14 days of moderate heat stress. Chlorophyll is usually the first port of call when analyzing plant health for both researchers and farmers as the “stay-green” trait often equates to healthiness visually, while leaf yellowing is associated to unhealthy. Unlike previous reports in a heat-sensitive tomato cultivar (Zhou et al., 2017), chlorophyll levels determined through PQ-SPAD parameter revealed maintenance of a high value in all plant groups regardless of the growth condition or ANE treatment applied. The fraction of light energy captured by PSII (Φ_II_) is an effective parameter that provides information on the nature of photoinhibition under abiotic stress. Indeed, a decline in Φ_II_ would be due to the inactivation of PSII reaction centers aimed at photoprotection (Mathur et al., 2014; Kuhlgert et al., 2016). Heat stress and ANE applications had some statistically significant effects on Φ_II_, indicating a minor modulation of PSII function. However, as observed before in other studies in tomato under moderate heat stress (Sato et al., 2000; Zhou et al., 2017), these differences in photosynthetic efficiency were not probably large enough to be the main factor related to the observed fruit set values. A decrease in Φ_II_ in untreated heat stressed plants was accompanied by a stimulation of Φ_NPQ_, a sensitive parameter used for monitoring thermal dissipation of excess light energy absorbed by PSII (Tiezt et al., 2017). Conversely, stressed plants treated with PSI-494 maintained similar Φ_NPQ_ levels to those recorded in unstressed plants. Consequently, these results suggest that this ANE biostimulant treatment has the potential to increase the energy available for photochemistry, which is a desirable physiological trait to improve crop yields under chronic mild stressful conditions (Malnoë, 2018).

### Impact of ANEs on Biochemical and Genetic Markers of Heat Stress Tolerance

It is important to highlight that it takes both pollination and fertilization to create a robust fruit set. If there is not an adequate amount of viable pollen, the male grain will not reach the stigma. However, timely pollination does not guarantee fruit set, as post-pollination processes such as pollen tube growth or fertilization are also heat sensitive (Peet et al., 1997; Erickson and Markhart, 2002). Therefore, we also evaluated whether the altered plant carbohydrate content observed in heat stressed plants was able to explain the stronger effect on fruit set provided by PSI-494. Sucrose is the primary end product of photosynthesis, which is translocated from source leaves to sink organs through phloem. Once it has reached those sinks, sucrose must be degraded into glucose and fructose (or their derivates) by sucrose synthase and invertase enzymes for various metabolic and biosynthetic processes (Ruan et al., 2010). Although untreated and ANE-treated plants had significantly lower sucrose content in leaf tissue after heat stress, this decrease was significantly mitigated with the application of PSI-494. In agreement with the results observed in a heat tolerant tomato line (Li et al., 2012), it is likely that more sucrose would be available for partitioning to reproductive organs in stressed plants treated with PSI-494. Flowers and young fruits have a high energy demand throughout their development and rely heavily on the source-to-sink flow for the supply of carbon resources (Borghi and Fernie, 2017; Shen et al., 2019). As previously reported in different tomato heat sensitive cultivars, our data specifically suggest that carbohydrate metabolism in flower tissue was disturbed under long-term moderate heat stress (Pressman et al., 2002, 2006; Firon et al., 2006; Sato et al., 2006). While soluble sugar levels were reduced in untreated stressed plants, this was more obvious for those plants treated with the ANE biostimulant extracted at low temperatures (C129). However, an improved ability to maintain glucose and fructose content and even increase sucrose in flowers of plants treated with PSI-494 was also observed. These differences in the accumulation pattern of soluble sugars may be an important factor in explaining the results observed in the fruit set for the heat stressed tomato plants. A relationship between an appropriate carbohydrate metabolism in flowers and young fruits and increased fruit set has been exhaustively described in the bibliography for heat tolerant tomato varieties (Pressman et al., 2002; Firon et al., 2006; Li et al., 2012), which supports the potential of specialized ANEs to strengthen inherent thermotolerance mechanisms.

Monitoring the expression levels of *HSP* genes can give important information on the capacity of reproductive organs to activate protective mechanisms required for thermotolerance. By re-establishing protein homeostasis, the induction of such chaperones can not only have a temporary survival effect but can allow increased efficiency of the fertilization process (Fragkostefanakis et al., 2015; Reddy et al., 2016). This research analyzed the expression levels of three particular *HSP* genes in flower tissues: *HSP101.1*, *HSP70.9*, and *HSP17.7C-Cl*. In this regard, the recent work of Fragkostefanakis et al. (2016), showed the important role of these three HSPs in the mechanism of thermotolerance of tomato pollen. When all three *HSP* gene expression levels were examined for the heat stressed plants each ANE biostimulant had a varied effect. Tomato plants treated with PSI-494 caused a statistically significant upregulation within *HSP101.1* and *HSP70.9*, while C129 induced a downregulation of both genes in the flowers of heat stressed plants. These concurrent changes were interesting as it has been described how HSP100 isoforms are essential components of plant thermotolerance implicated in protein disaggregation, an activity that is complemented for refolding by cooperation with HSP70 isoforms (Seyffer et al., 2012; Fragkostefanakis et al., 2015; Mogk et al., 2015). Although the increase of *HSP17.7C-Cl* gene expression in flowers of stressed plants treated with PSI-494 was only statistically significant at 80% confidence interval, it is interesting to emphasize the potential biological significance of these results. As mentioned for HSP70, a collaborative mechanism between HSP101 and sHPSs for reverting irreversible aggregation of heat-sensitive proteins has also been reported (McLoughlin et al., 2016). Moreover, the overexpression of sHSP in tomato anthers and young fruits has been proposed as a significant contributor factor to heat tolerance (Giorno et al., 2010; Li et al., 2012). Therefore, the differential effect of ANEs on the expression levels of some relevant *HSP* genes in flower tissues may support a potential mode of action in the induction of thermotolerance.

### Summary and Perspectives

Modern day agriculture is becoming more unpredictable due to climate change and the subsequent increase in abiotic stresses such as heat. ANE biostimulants can be a viable solution in creating more sustainable and environmentally acceptable agricultural practices. One of the current challenges is in acquiring acceptance among the agricultural community. This can only be achieved through communicating extensive research into defined mode of action and demonstrating the robustness of these crop inputs. Overall, our data indicate that treatment with one specialized ANE (PSI-494) could represent a potential tool for farmers to alleviate the damage of long periods of moderate heat stress at the reproductive stage leading to enhanced fruit set. Physicochemical characteristics of ANE biostimulants are derived from their processing conditions and appear to be related to their performance in enhancing fruit set during heat stress. This has been demonstrated by an increased flower number, improved pollen viability, enhanced carbohydrate metabolism, and *HSPs* gene expression in reproductive organs before fertilization.

## Data Availability Statement

All datasets generated for this study are included in the article.

## Author Contributions

OG and SO’C secured funding and supervised the work. NC, OG, and SO’C conceived and designed the experiments. NC, OG, and ŁŁ performed the experiments. NC, OG, ŁŁ, and SO’C analyzed the data and wrote the article. All authors contributed to the article and approved the submitted version.

## Conflict of Interest

While Brandon Bioscience provided support in the form of salary for the author ŁŁ, this company did not have any additional role in the study design, data collection and analysis, decision to publish, or preparation of the manuscript.

The remaining authors declare that the research was conducted in the absence of any commercial or financial relationships that could be construed as a potential conflict of interest.
